# Mobile phone-based interventions for smoking cessation

**DOI:** 10.1590/S1516-31802010000200014

**Published:** 2010-03-04

## Abstract

**BACKGROUND:**

Innovative effective smoking cessation interventions are required to appeal to those who are not accessing traditional cessation services. Mobile phones are widely used and are now well integrated into the daily lives of many, particularly young adults. Mobile phones are a potential medium for the delivery of health programmes such as smoking cessation.

**OBJECTIVES:**

To determine whether mobile phone-based interventions are effective at helping people who smoke, to quit.

**SEARCH STRATEGY:**

We searched MEDLINE, EMBASE, Cinahl, PsycINFO, The Cochrane Library, the National Research Register and the ClinicalTrials register, with no restrictions placed on language or publication date.

**SELECTION CRITERIA:**

We included randomized or quasi-randomized trials. Participants were smokers of any age who wanted to quit. Studies were those examining any type of mobile phone-based intervention. This included any intervention aimed at mobile phone users, based around delivery via mobile phone, and using any functions or applications that can be used or sent via a mobile phone.

**DATA COLLECTION AND ANALYSIS:**

Information on the specified quality criteria and methodological details was extracted using a standardised form. Participants who dropped out of the trials or were lost to follow up were considered to be smoking. Meta-analysis of the included studies was undertaken using the Mantel-Haenszel Risk Ratio fixed-effect method provided that there was no evidence of substantial statistical heterogeneity as assessed by the I2 statistic. Where meta-analysis was not possible, summary and descriptive statistics are presented.

**MAIN RESULTS:**

Four studies were excluded as they were small non-randomized feasibility studies, and two studies were excluded because follow up was less than six months. Four trials (reported in five papers) are included: a text message programme in New Zealand; a text message programme in the UK; and an Internet and mobile phone programme involving two different groups in Norway. The different types of interventions are analysed separately. When combined by meta-analysis the text message programme trials showed a significant increase in short-term self-reported quitting (RR 2.18, 95% CI 1.80 to 2.65). However, there was considerable heterogeneity in long-term outcomes, with the much larger trial having problems with misclassification of outcomes; therefore these data were not combined. When the data from the Internet and mobile phone programmes were pooled we found statistically significant increases in both short and long-term self-reported quitting (RR 2.03, 95% CI 1.40 to 2.94).

**AUTHORS’ CONCLUSIONS:**

The current evidence shows no effect of mobile phone-based smoking cessation interventions on long-term outcome. While short-term results are positive, more rigorous studies of the long-term effects of mobile phone-based smoking cessation interventions are needed.

**FURTHER INFORMATION:** Centro Cochrane do Brasil Rua Pedro de Toledo, 598 Vila Clementino — São Paulo (SP) — Brasil CEP 04039-001 Tel. (+55 11) 5579-0469/5575-2970 http://ww.centrocohranedobrasil.org.br/

This section was edited under the responsibility of the Brazilian Cochrane Center

The full review is available (free access) from: http://cochrane.bvsalud.org/portal/php/index.php?lang=pt

## COMMENTS

Smoking cessation is one of the most important interventions regarding long-term reduction of morbidity and mortality under a variety of conditions. Many strategies have been tried and there is growing interest in using modern technology to achieve this goal.

In a recently published article,^1^ it was sought to determine whether studies using mobile phone-based interventions would be of relevance in smoking cessation. After a thorough search in medical the literature, four studies were found to be eligible for analysis. It was concluded that mobile phone-based interventions may be useful over the short term, but there is no evidence of their efficacy over the long term.

This result highlights the growing use of available modern technology in medical care and offers some insight into how to design new trials in order to explore its potential.
